# Immunomodulatory effects of exercise in cancer prevention and adjuvant therapy: a narrative review

**DOI:** 10.3389/fphys.2023.1292580

**Published:** 2024-01-04

**Authors:** Da-wei Lyu

**Affiliations:** Physical Education and Health School, East China Jiaotong University, Nanchang, Jiangxi, China

**Keywords:** exercise, immunomodulation, cancer prevention, physical activity, anticancer

## Abstract

Successful application of cancer immunotherapy has rekindled hope in cancer patients. However, a number of patients are unresponsive to immunotherapy and related treatments. This unresponsiveness in cancer patients toward different treatment regimens can be mainly attributed to severe immune dysfunction in such patients. Several reports indicate that physical exercise can significantly lead to improved cancer patient outcomes. Since exercise gets immense response from the immune system, it can be utilized to improve immune function. Leukocytes with enhanced functions are substantially mobilized into the circulation by a single bout of intense physical exercise. Chronic physical exercise results in greater muscle endurance and strength and improved cardiorespiratory function. This exercise regime is also useful in improving T-cell abundance and reducing dysfunctional T cells. The current available data strongly justify for future clinical trials to investigate physical exercise use as an adjuvant in cancer therapy; however, optimal parameters using exercise for a defined outcome are yet to be established. The components of the immune system associate with almost every tumorigenesis step. The inter-relationship between inflammation, cancer, and innate immunity has recently gained acceptance; however, the underlying cellular and molecular mechanisms behind this relationship are yet to be solved. Several studies suggest physical exercise–mediated induction of immune cells to elicit anti-tumorigenic effects. This indicates the potential of exercising in modulating the behavior of immune cells to inhibit tumor progression. However, further mechanistic details behind physical exercise–driven immunomodulation and anticancer effects have to be determined. This review aims to summarize and discuss the association between physical exercise and immune function modulation and the potential of exercise as an adjuvant therapy in cancer prevention and treatment.

## 1 Introduction

Multiple human studies have established the beneficial effects of exercise in lowering the risk of cancer development (Amiri et al., 2023; Pelzer et al., 2023; Rogers et al., 2023). Leisure-time exercise reduces 13 cancer types as suggested by an epidemiological study (Moore et al., 2016). Several reports indicate the positive effect of physical activity in reducing the recurrent risk for prostate, colon, and breast cancers (Kenfield et al., 2011; Brown et al., 2023; Rogers et al., 2023). Similarly, esophageal, renal, uterine, and stomach cancers can be reduced through exercise (Behrens and Leitzmann, 2013; Kitson et al., 2022; Lavery et al., 2023). Some of the studies in humans show a positive influence of exercising on habit formation, as people under a regime of physical activity are also more likely to lead a healthy lifestyle (Kenfield et al., 2011; Amiri et al., 2023). The general consensus is that exercise is a critical lifestyle habit that has the potential to lower the risk of different cancer types. Preclinical studies on exercise have concluded on the protective effect of exercise against cancer (Jia et al., 2021; Jee et al., 2022). In rodents, physical activity interventions, such as swimming, treadmill running, and wheel running, show reduced tumor growth, incidence, and metastasis across a variety of chemically and genetically induced tumor models (Jia et al., 2021; Jee et al., 2022). In humans, differential sensitivity to physical activity has been observed based on the genetic background of the cancer type (Jones et al., 2016). Exercise training shows no regulation in a mouse model of breast cancer with a p53^−/−^ MMTV-Wnt background, suggesting a stronger initiating driver mutation in this tumor model (Colbert et al., 2009). Similarly in humans, premenopausal women show lesser response to physical activity–mediated benefits against breast cancer than postmenopausal women (Jones et al., 2016). Altogether, despite such varied responses, regular physical activity in general can reduce cancer risk and control the growth of tumors, irrespective of the type of cancer diagnosis.

The mechanisms underlying exercise-mediated beneficial effects in cancer progression and prevention are probably multifaceted and complex (Neilson et al., 2009; Emery et al., 2022). Inflammation plays an important role in cancer progression, and physical activity has been shown to induce anti-inflammatory response (Pierce et al., 2009). Inflammation has been linked to various steps involved in tumorigenesis (Coussens and Werb, 2002), and exercise has been reported to reduce inflammation (Petersen and Pedersen, 1985). The optimal dosage and mode of physical activity are not very clear from the available literature; however, it is generally agreed that as compared to sedentary individuals, moderate [3–5.9 metabolic equivalents (METs).h/week] to higher (<6 METs.h/week) exercising individuals show cancer risk reduction by at least 10%–20% (Patel et al., 2019; Friedenreich et al., 2021). Besides, other factors like starting to exercise early and healthy lifestyle habits seem to contribute to lowering the risk of cancer (Rezende et al., 2018; McTiernan et al., 2019). Several mechanisms might be involved in exerting cancer-preventative effects, and the importance of each individual contribution may vary depending on various other factors and the cancer type. Among the different mechanisms, stimulating the immune system can be an effective way in the prevention of initiation, progression, and spread of primary tumors (Emery et al., 2022). Immune cells can help in tumor antigen-specific recognition and destruction of cancer cells. Studies in both human and animal models suggest that crosstalk between tissues during physical activity causes immune system modulation and provides protection against cancer (Lucia and Ramirez, 2016; Febbraio, 2017).

**FIGURE 1:**
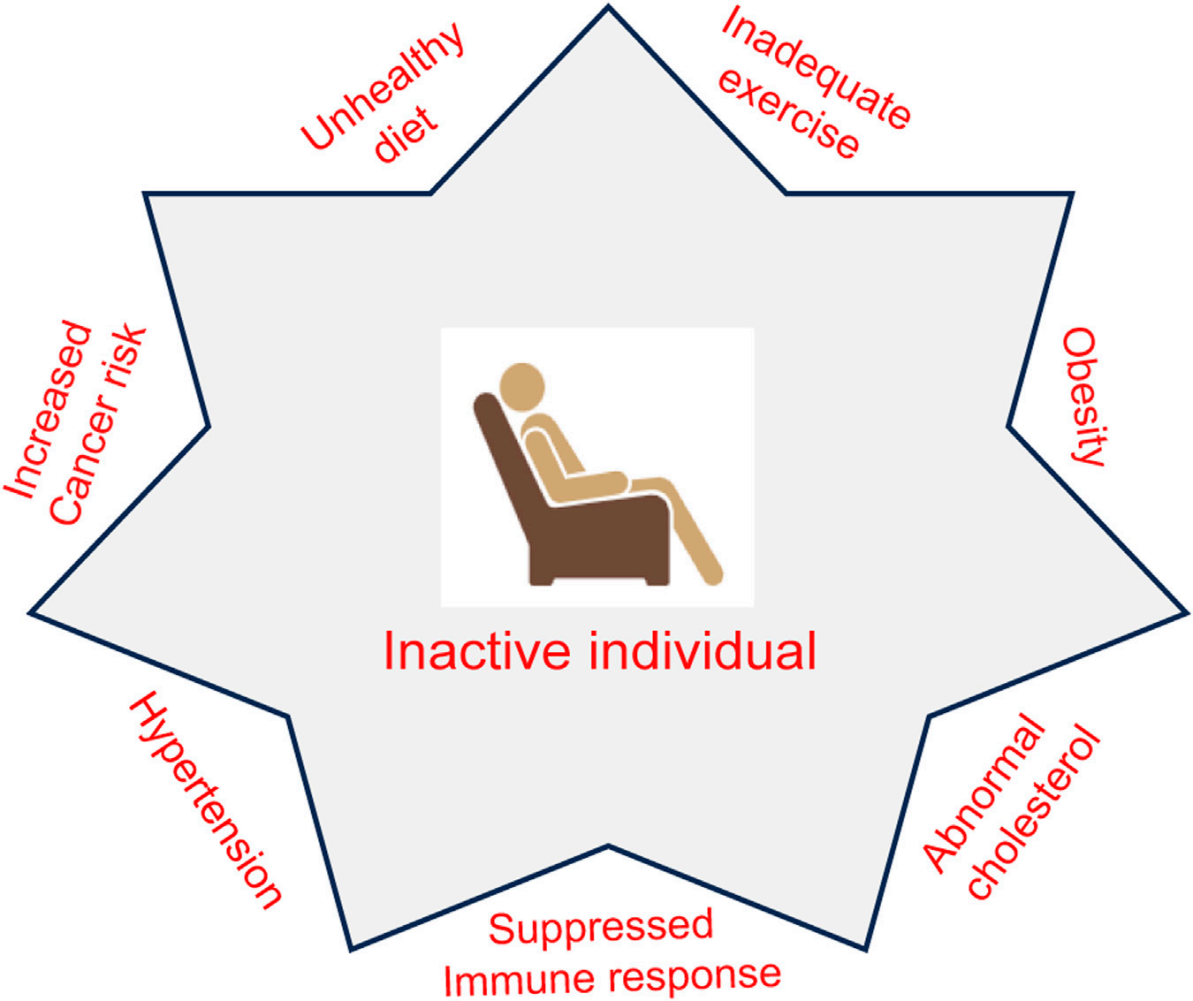
Association of an inactive lifestyle with pathophysiological conditions. The lack of physical activity may lead to a risk for conditions such as obesity, abnormal cholesterol, hypertension, and a suppressed immune system. Inadequate exercise along with unhealthy diet increases the risk for cancer development.

Exercise training includes repetitive rounds of physical activity challenging the homeostasis of the entire body, causing wider adaptations within the organ systems, tissues, and cells (Hargreaves, 1985; Gabriel and Zierath, 2017). Studies have been carried out to understand metabolic and biochemical adaptations in the adipose tissue, heart, skeletal muscle, and vasculature, but very little is known about such adaptations in tumor tissues (Guan and Yan, 2022). However, physical activity has been the proposed strategy for favorable results in cancer patients. A sedentary lifestyle, on the other hand, is associated with increased risk for different pathophysiological conditions as well as cancer (Figure 1). Although multiple studies report the heterogeneity of physical activity–associated intervention in cancer patient care, such interventions in general can be linked to achieve positive outcomes in physiological measures (e.g., physical function, cardiopulmonary fitness, body composition) (Sasso et al., 2015; Carter et al., 2021). Notwithstanding the importance of these parameters to cancer patients, a recent finding indicates that tumor biology can be more directly controlled by exercise, thereby improving the clinical outcome (Salamon et al., 2023). The underlying mechanistic insights are crucial if exercise is to be considered as part of “cancer medicine.” Once such knowledge is established, a more personalized approach can be taken in training exercises to cancer patients instead of the usual “one size fits all” approach since different modes, intensities, and amount of exercise can lead to different molecular outcomes in cancer. This review aims to elaborate on the findings that highlight the underlying mechanism of immune modulation through exercise that results in reducing the risk of cancer and explores the potential use of exercise as an adjuvant therapy during cancer treatment.

## 2 Molecular mechanisms underlying the benefits of exercise in cancer

It is generally agreed that a deeper understanding into the associations between exercise and cancer prevention would be useful in providing precise and adequate dosage of physical activity (McTiernan, 2008). Several mechanisms have been postulated that include explanations related to metabolism, sex hormones, oxidative stress, immune function, DNA damage repair, inflammation, adiposity, and so on (Jurdana, 2021). The existing literature does not provide enough support to any one of the mechanisms, except for immune system modulation and activation, which will be dealt with in detail in this review, while outlining the other proposed mechanisms. It is more useful to observe a higher number of individuals in relation to their physical activity at pre-defined stages, until some of them develop cancer, given the fact that a long latent period is associated with cancer development. Since it is difficult to follow this approach, the alternative approach will be to measure the molecular pathways–associated surrogate biomarkers that have been linked to the association between exercise and prevention of cancer risk (Baker and Kramer, 2020).

### 2.1 Hormones

Increased levels of estrogens and androgens in women cause an increase in breast cancer risk (Tin Tin et al., 2021). In addition, women with increased levels of estrogen show an elevated risk for developing endometrial cancer (Friedenreich et al., 2020). Prostate cancer survival is significantly improved following anti-androgen therapy in men (Hafron et al., 2023). The overall disease incidence is also improved using such therapeutic strategies as a preventative measure. The positive influence of exercise on endogenous sex hormone levels and overall menstrual cycle function in young women can be a potential barrier to developing breast cancer (McTiernan et al., 2019). Postmenopausal women show lower serum levels of androgens, estradiol, and estrone following exercise (Chan et al., 2007). There is a close association between body composition and the positive effect of exercise in postmenopausal women as their main source of estrogen is androgen precursors mainly in the peripheral adipose tissues (Chan et al., 2007). As compared to active women, sedentary women with a lower level of physical activity show higher serum concentrations of estradiol and estrone, with lower sex hormone–binding globulin levels. In women losing body fat, physical activity results in significantly reduced levels of both estrogens and androgens (Chan et al., 2007). These results confirm the benefits of both reduced body fat and increased exercise in reducing serum sex hormone levels and providing protection against breast cancer. The association between sex hormones and exercise in men has not been well studied; however, in athletes, significantly lower levels of testosterone have been reported (Moore et al., 2021). Excess androgen levels have been suggested to increase prostate cancer risk, while at the same time, finasteride, a testosterone-reducing medication, lowers prostate cancer incidence (Hafron et al., 2023). However, there is also the report of finasteride increasing the risk of high-grade prostate cancers (Hafron et al., 2023). Since a low level of testosterone is associated with obesity in men, the effect of exercise on testosterone gets further complicated (Loh et al., 2022). Although the link between the risk of prostate cancer and blood concentrations of sex hormones is not well established, an epidemiological study reported that prostate cancer survival is associated with low dihydrotestosterone (DHT) levels (Lundgren et al., 2020). Furthermore, prostate cancer aggressiveness is found associated with low DHT levels. Therefore, an increase in DHT levels through physical activity could achieve potential benefits in prostate cancer treatment.

### 2.2 Gastrointestinal tract

The gastrointestinal tract (GIT) in humans is colonized by microbial cells in huge numbers, and multiple studies have validated the significance of the composition of gut microbiota in different pathophysiological processes that include both gastrointestinal locally and other cancers distally (such as breast, pancreatic, prostate, head and neck, esophageal, and so on) (Goodman and Gardner, 2018; Zyoud et al., 2022). Different risk factors, such as inflammation, aging, an unhealthy diet, and smoking, can cause dysbiosis or microbial imbalance, thereby increasing the risk of neoplastic transformation (Zyoud et al., 2022). Gut microbiota are involved in providing nutrition, regulating the development of epithelia, and affecting the immune response. Besides, an improvement in microbial diversity has been found to be associated with improvement in the immune system and overall health status (Johnson, 2020). Different factors can modulate the gut microbiota, as demonstrated by multiple studies (Goodman and Gardner, 2018; Johnson, 2020; Zyoud et al., 2022). Zhang et al. (2022) suggest that physical activity improves microbial diversity and composition and influences useful metabolite production by gut bacteria. Aerobic exercise has a positive influence on the gut by improving colon motility and reducing transient stool time, causing a reduction in pathogen contact time with gastrointestinal mucosa (Clauss et al., 2021). The intestinal integrity is protected through exercise-induced reduction in inflammation and prostaglandin production. This mechanism seems to provide the rationale behind exercise-mediated protection against several diseases such as inflammatory diseases, colon cancer, and diverticulosis (Clauss et al., 2021). In addition, physical activity causes an increase in immunoglobulin production and elevated levels of short-chain fatty acids such as increased butyrate levels, which show anti-inflammatory and anti-carcinogenic properties (Matsumoto et al., 2008). Physical activity can alter both the diversity and composition of microbiota by preventing obesity and related pathologies (Clauss et al., 2021). Thus, regular exercise can be a critical factor that results in altered microbial composition, benefiting the host by stimulating useful metabolite production from gut bacteria that provides protection against gastrointestinal pathologies and colon cancer.

### 2.3 DNA damage repair

The expression of genes has been found to be influenced by physical activity; however, it is yet to be established as to how such genetic and epigenetic changes influence the risk of cancer (Friedenreich et al., 2021). A trial with men in the Gene Expression Modulation by Intervention with Nutrition and Lifestyle (GEMINAL) study found that regular lifestyle interventions and physical activity downregulated members of the oncogenic Rat sarcoma (RAS) family (RAB8A, RAB14, and RAN) in prostate cancer (Ornish et al., 2008). RAS-related nuclear protein (RAN) functions as a co-activator of androgen receptors within the prostate tissue (Ornish et al., 2008). A study on prostate cancer revealed alterations in the expression of >180 genes when sedentary men were compared with men who were physically active (Magbanua et al., 2014). The genes particularly affected were those involved in DNA damage repair like BRCA1 and BRCA2, in the histone deacetylase pathway, and in the cell cycle. Other human and rodent studies on cellular repair process markers reported the upregulation of p53 through physical activity that induces the repair or destruction of damaged cells (Wang et al., 2009; Sharafi and Rahimi, 2012). In men with active surveillance for prostate cancer, the telomere length was established as a prognostic marker (Ornish et al., 2013). Men on a healthy diet and physically active regime had an increased telomere length as compared to inactive men, and this was associated with a reduction in prostate-specific antigen (PSA) progression (Ornish et al., 2013).

### 2.4 Adiposity and inflammation

Several different types of cancer risk have been found to be associated with adiposity-related chronic inflammation (Divella et al., 2016; Wu et al., 2021). Adipose tissue is involved in the production and release of anti-inflammatory adiponectin and pro-inflammatory cytokines–like tumor necrosis factor α (TNF-α), C-reactive protein (CRP), interleukin 6 (IL-6), and leptin. Skeletal muscle cells through resistance training exercise can be made to produce different families of anti-inflammatory myokines that lead to increased levels of adiponectin and reduced secretion of pro-inflammatory TNF-α, IL-6, and leptin (de Assis and Murawska-Cialowicz, 2023). Pro-inflammatory cytokines like leptin have been found to be associated with multiple different cancer developments, thereby establishing a link between cancer and obesity (Moodi et al., 2021). Elevated leptin levels in humans are directly associated with excessive adipose tissue, and this is lower in men than in women even when the total body fat gets adjusted (Ramel et al., 2010; Obradovic et al., 2021). One probable reason for this difference is the sex hormone–mediated leptin expression regulation; accordingly, it was shown that leptin levels are increased by estrogens while leptin levels are decreased by testosterone (Cheng et al., 2022). Besides, leptin can cause enhanced expression of angiogenic (vascular endothelial growth factor, VEGF), inflammatory factors (TNF-α, IL-6), and anti-apoptotic proteins by acting as a mitogen. Interestingly, resistance exercise exerts an anti-inflammatory effect by lowering leptin levels (Divella et al., 2016; Nuri et al., 2016; de Assis and Murawska-Cialowicz, 2023). Studies on colon cancer and breast cancer have provided evidence to link leptin with neoplastic processes; however, limited information is available on this association in other types of cancer (Nuri et al., 2016; Lin and Hsiao, 2021; de Assis and Murawska-Cialowicz, 2023). Furthermore, adiponectin levels in adipose tissue show anti-inflammatory effects that can prevent obesity-related inflammation and increased risk of cancer (Divella et al., 2016). Insulin sensitivity is found to be associated with plasma concentrations of adiponectin that in turn can be linked to metabolic disorders and cancer risk (Wu et al., 2021). It has been postulated that physical activity–induced insulin sensitivity improvement is caused by regulating plasma concentrations of adiponectin. Moreover, high levels of serum leptin and low levels of serum adiponectin are independently associated with cancer metastasis risk (Divella et al., 2016; Wu et al., 2021). As sedentary lifestyle is found associated with obesity/excess weight gain, both being critical risk factors for developing cancer, regular exercise can be useful for cancer prevention by lowering adiposity (Divella et al., 2016).

### 2.5 Metabolism

Physical activity contributes to anti-cancerous effects by altering the cancer cell energy metabolism since exercise, in general, has the potential to perturb different physiological processes controlled by integrated homeostatic circuits (Pedersen et al., 2015). Extrinsic factors in tumors, constituting the extracellular region, get altered through physical activity, thereby changing the energy metabolism of tumor cells (Pedersen et al., 2015). Thus, exercise can alter energy metabolism in tumors, reducing their rate of growth, by decreasing the functioning of mitochondria in tumor models. Besides, in a colorectal murine cancer model, mice with aerobic exercise training showed modified carbon metabolism along with a delay in tumor development (Lu et al., 2018). Such analysis shows the physical activity–dependent downregulation of the tricarboxylic acid cycle (TCA) cycle, apart from glutamate and succinate (Lu et al., 2018). In addition, it was found that physical activity causes modulations of nucleotide precursors. Physical activity increased ADP-ribose and adenine, with decreased hypoxanthine and inosine monophosphate during purine metabolism, while there was a decrease in UDP, uracil, and ureidosuccinic acid and an increase in dihydrothymine during pyrimidine metabolism (Lu et al., 2018). Exercise also provides for β-oxidation of substrates by reducing the levels of stearoyl carnitine, acylcarnitines, and L-palmitoyl carnitine, which play important roles in the oxidation of lipids. Also, in such individuals, phosphocreatine gets significantly reduced, which is critical in high energy–consuming metabolic conditions (Lu et al., 2018). The cell metabolism by-products—TCA cycle metabolites—are a critical ingredient for macromolecule biosynthesis like that of lipids, proteins, and nucleotides. They are also important factors for cell signaling functions, which include tumor development (Chandel et al., 1998). Additionally, the immune response is modulated by metabolites like succinate, fumarate, α-ketoglutarate, and acetyl-CoA that can alter the immune response (Liu et al., 1996). These metabolites also regulate the maintenance of stem cell pluripotency and lymphangiogenesis (Wong et al., 2017). Glutamate is generated via glutamine metabolism, which also acts as a TCA cycle carbon source and aids in the synthesis of fatty acids. This is brought about by isocitrate dehydrogenase via α-ketoglutarate reductive carboxylation to produce citrate by using NADPH (Carey et al., 2015). The complexity associated with the metabolism of tumor energy is revealed through the regulation of metabolism and reductive carboxylation by TCA cycle intermediates. Nonetheless, compared to sedentary individuals, physically active individuals can significantly overcome tumor metabolism–associated complexities that result in delaying the development of tumors. However, it is important to assess the tumor development stage and the tumor subtype before ascertaining the benefit of physical activity in tumor regression. Therefore, irrespective of the vast amount of literature supporting the positive impact of exercise in controlling and regressing the development and progression of cancer, future studies further have to clearly validate the underlying mechanisms of exercise-associated anti-cancerous effects in this regard.

## 3 Immunity

**FIGURE 2:**
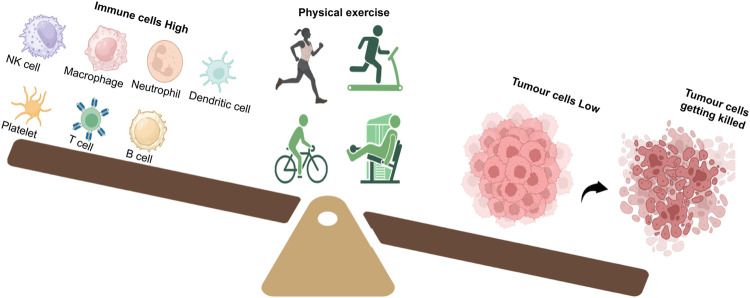
Exercise tilts the balance in favor of a strong immune response. A good regime of exercise in cancer patients improves the number and function of immune cells such as NK cells, macrophages, neutrophils, dendritic cells, platelets, T cells, and B cells. Activation of the immune system results in tumor cell killing and reduction.

Our body’s immune system plays a critical part in cancer risk reduction through the components of the innate and/or acquired immune system by recognizing and eliminating abnormal tumor cells (Jakobisiak et al., 2003). One of the accepted hypotheses behind this observation is that exercise causes improvement in the elevation and functioning of several different immune cells, which include natural killer (NK) cells, macrophages, neutrophils, dendritic cells, platelets, and T and B cells, all of which have been found to have tumor-suppressive roles (Figure 2). An acute elevation in the levels of different immune function components such as lymphocytes, monocytes, neutrophils, and eosinophils has been observed following bouts of physical activity (Nieman and Pence, 2020). Moderate exercise (≤9 MET.h/week) resulted in immune response enhancement, while high-intensity or overtraining exercise (<24 MET.h/week) resulted in suppression of the immune system (McTiernan, 2008). However, differences between non-exercisers and exercisers in terms of their immune function improvement could not be noted across all cross-sectional studies and large longitudinal studies, clearly outlining the various parameters like the onset of tumor and rate of tumor growth as outlined by McTiernan (2008).

### 3.1 Immunological profile of tumor cells

Recognition and elimination of tumor cells by the host immune system is one of the most potent therapeutic strategies against cancer, and accordingly, cancer prognosis has been linked closely with tumor cell profile of immunological components. Thus, it is an accepted notion that the higher the number of infiltrating cytotoxic T cells and NK cells in cancer patient tumors, the better the associated prognosis (Strauss and Thomas, 2010; Lee and Oh, 2021). Tumor cells have devised ways to paralyze cytotoxic immune cell infiltration to escape elimination by immune cells. Tumor cells can control immune cell cytotoxicity by enhanced expression of inhibitory receptor ligands (such as B7.1, PD-L1, etc.) or suppress the immune cell functioning by secreting immunosuppressive agents such as transforming growth factor β (TGF-β) (Sharma and Allison, 2015). It was shown in a study that physical activity like wheel running can cause redistribution and mobilization of cytotoxic immune cells, resulting in tumor growth regression (Pedersen et al., 2015). The tumor cell immunological profile is critical in the redistribution of killer immune cells toward cancerous cells (Nieman and Pence, 2020). In fact, physical activity elevates the levels of receptor ligands meant for NK cell activation, blocking ligands for immune checkpoints and immune-attracting chemokines, and there are studies that reveal the mechanism behind such tumor-enhanced immunogenic responses (Tasdemir et al., 2016; Muller et al., 2017). The type-I interferon pathway can play chemo- and immune-attractant roles in different pathways, causing improvement in immune recognition, and it may as well play an important role here (Muller et al., 2017). It is also noteworthy to mention that cellular senescence induces tumor immunogenicity through enhanced cytotoxic T-cell and NK cell infiltrations and type-I interferon (Tasdemir et al., 2016). It is suggested that cellular senescence is decreased by physical activity depending on the telomere length regulation in immune cells (Ornish et al., 2013). Physical activity–driven control of tumor immunogenicity may also be brought about by metabolic byproducts (Angelin et al., 2017). Accumulation of higher lactate levels occurs within cancerous cells owing to modulated tumor cell metabolism–mediated enhanced aerobic glycolysis levels. Functioning of immune cells, such as cytotoxic T cells, gets inhibited through elevated lactate levels (Angelin et al., 2017). Physical activity can relieve this form of immunosuppression by lowering the lactate levels in tumor cells (Aveseh et al., 2015). There are profound clinical implications mediated by this regulation. Tumor levels of Lactate dehydrogenase are a measure to classify melanoma cancer patients into low and high risk, and elevated Lactate dehydrogenase levels have been linked to poor prognosis (Hersey et al., 2009). Overall, it is suggested that immune infiltration triggering and immunosuppressive metabolite alleviating events promote elevated tumor immunogenicity in exercising individuals.

### 3.2 Physical activity–dependent modulation of immune cells

**FIGURE 3:**
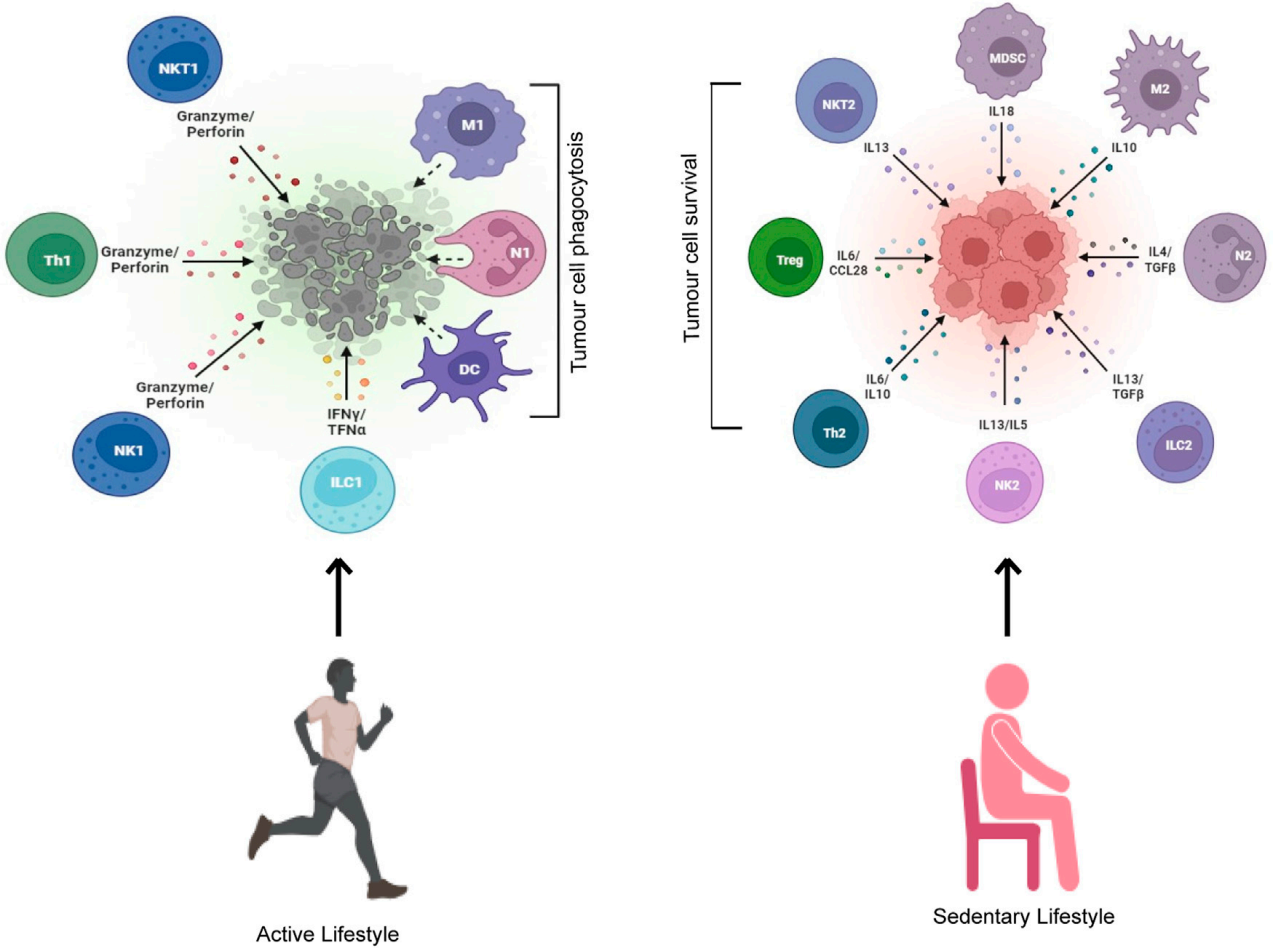
An active but not sedentary lifestyle induces tumor cell killing. Exercising individuals leading an active lifestyle get their immune system activated with enhanced cytotoxicity toward tumor cells. A sedentary lifestyle, on the other hand, activates a pro-survival immune response that leads to increased tumor cell survival.

The innate immune system is positively influenced by training exercise. Remobilization of killer immune cells into circulation during physical activity occurs through stress-mediated adrenergic signaling (Tong et al., 2022). This mobilization causes the recruitment of the existing immune cell pool but does not necessarily result in the production of newer immune cells (Tong et al., 2022). Acute and chronic aerobic physical exercise results in significant immunomodulation in terms of redistribution, function, and activity of immune cells toward tumor cells; sedentary lifestyle, on the other hand, results in immunomodulation toward tumor cell survival (Figure 3). The duration and intensity of physical activity also influence immune cell redistribution into circulation (Salimans et al., 2022; Tong et al., 2022). In animal models, physical activity resulted in a decreased number of immunosuppressive cells and an increased number of functional effector cells (Hagar et al., 2019). Here, we will discuss in detail physical activity–driven immunomodulation through the impact on multiple different immune cells.

#### 3.2.1 Natural killer cells

During exercise, NK cells get quickly mobilized into the circulation, acting as the best responders among the immune cells (Tong et al., 2022). In several different cancer settings, NK cell modulation and activation have been known to be an effective mode of immunotherapy (Chester et al., 2015; Tong et al., 2022). NK cells exert a cytotoxic effect on tumor cells by recognizing them and promoting elevated response of T cells via the production of chemokines and cytokines, enhancing overall NK cell–mediated tumor cell cytotoxicity (Chester et al., 2015). Unlike their healthy counterparts, transformed tumor cells are often found to express ligands like major histocompatibility complex (MHC) class 1 chain–related proteins A and B (MICA and MICB) (Garcia-Cuesta et al., 2015). Cytotoxicity receptor NKG2D uses MICA and MICB as ligands and destroys cancer cells (Lanier, 2015). Inhibitory receptors that engage human leukocyte antigen (HLA)–like cytotoxic immunoglobulin receptors result in normal tissue protection by the downregulation of cytotoxicity. The cytotoxic effect of NK cells is reduced in the presence of soluble MICA and tumor cell–expressed HLA class I molecules (Groh et al., 2002). In hematologic malignancies, enhanced cancer cell expression of HLA class I results in defective cytotoxic effect of NK cells. In patients with chronic lymphocytic leukemia (CLL), acute myeloid leukemia (AML), and multiple myeloma, NK cells show reduced expression of NKG2D, which in turn is linked to enhanced levels of MICA and MICB, thereby causing anergy in NK cells (Xing and Ferrari de Andrade, 2020). The cytotoxic effect of NK cells is influenced by different available malignancy drugs. Pomalidomide and lenalidomide lead to enhanced NK cell proliferation and cytotoxicity (Wu et al., 2008). Bortezomib, particularly in a combinatorial approach using either glycogen synthase kinase-3β (GSK3β) inhibitor or radiation, causes the downregulation of HLA molecules and upregulation of NKG2D expression in cancer cells (Lee et al., 2018). During a single aerobic physical activity session, the blood NK cell composition and number get modulated, which return to their normal levels briefly after the end of an exercise session (Evans et al., 2015; Tong et al., 2022). During physical activity, cells are mobilized into the blood from the tissues and then again remobilized from the blood to the tissues following the end of exercise. This explains early physical activity–associated NK cell activity (NKCA) that shows an increase since there is an increase in the ratio of NK cells to tumor cells (Tong et al., 2022). Following the adjustment in absolute NK cell numbers for physical activity–driven increase, no change occurs in NKCA that are HLA-negative in myeloma and lymphoma cell lines when compared to that in healthy individuals (Bigley et al., 2015; Tong et al., 2022). During the egress following physical activity, NK cells in healthy adult blood show enhanced NKCA against HLA-positive but not HLA-negative cell lines. Such cell killing properties have been linked with modulations in activating (NKG2C) and inhibiting (CD158b) receptors, resulting in enhanced cytotoxic effect on tumor cells during and after physical activity.

#### 3.2.2 Macrophages

Macrophages are critical in the regulation of tumor metastasis. Several circulating tumor cells (CTCs) are diminished by M1 macrophages, while tumor metastasis is promoted by M2 macrophages. Physical activity increased the cytotoxic effect of macrophages and suppressed macrophage polarity to M2 (Goh et al., 2012). Training in short-term moderate physical activity enhanced anticancer cytotoxicity of macrophages and reduced metastases in an animal lung cancer model (Murphy et al., 1985). A recent work on a breast cancer model indicated that physical activity decreased the polarization of M2 macrophages by blocking the JAK-STAT pathway (Kim et al., 2020). Chemokine CC motif chemokine ligand 22 (CCL22) is released by M2 macrophages, which attracts Tregs-expressing CC chemokine receptor 4 (CCR4) to the CCL22 gradient in circulation, resulting in Treg recruitment (Kim et al., 2020). Physical activity significantly contributed to reduced expression of CCL22 mRNA, resulting in Treg recruitment reduction in M2 macrophages. This resulted in a delay in metastasis and progression of breast cancer in a mouse model (Goh et al., 2013). Similarly, the transcript levels of markers like Arg, CD206, and CCL22 that are M2 macrophage related are found reduced in physically active mice (McClellan et al., 2014).

#### 3.2.3 Neutrophils

Neutrophils aid the host defense mechanism through intracellular killing and phagocytosis of pathogens, immunomodulatory chemokine and cytokine release, antimicrobial peptide release, and DNA trap (histone-bound) formation, also called extracellular neutrophil traps (Yang et al., 2017). Neutrophils in circulation stimulate tumor cell aggregation, improving the CTC survival rate (Szczerba et al., 2019). Tumor metastasis is increased by neutrophil-released extracellular traps (NETs), the formation of which can be inhibited through exercise (Shi et al., 2020). In old individuals, the primary function of neutrophils is either defective or decreased, resulting in enhanced infection risk and improper tissue insult resurrection (Butcher et al., 2000). The literature supporting physical activity–mediated functions of neutrophils is ever increasing. In older adults with rheumatoid arthritis, interval style walking for 10 weeks could be linked to improved chemotactic and phagocytic capacity of neutrophils toward bacterial infections (Bartlett et al., 2018). Older healthy adults engaged in walking 10,000 steps per day showed greater chemotactic activity of neutrophils toward pathogens than older adults walking 5,000 steps a day (Bartlett et al., 2016). Weeks of strength training or an acute aerobic exercise session resulted in improved phagocytosis, chemotaxis, mitochondrial function, and extracellular neutrophil trap formation in young healthy adults (Syu et al., 2012). Both hematologic and solid tumors show poor outcomes during neutrophil dysfunction. Cancer outcomes can be significantly improved if physical activity is used as an adjuvant therapy in the improvement of neutrophil, NK cell, and T-cell functions.

#### 3.2.4 Dendritic cells

Dendritic cells (DCs) play a critical role in the control and elimination of tumor cells. A study on human exercise showed that physical activity results in the enhancement of peripheral blood DCs (LaVoy et al., 2015a). Furthermore, physical activity results in the upregulation of DC-associated IL-12 and MHC II expression in animal models (Baker et al., 2022). A study investigating the mobilized DC subpopulation composition owing to acute aerobic physical activity found that plasmacytoid DCs are preferentially mobilized into peripheral circulation following exercise, which results in improved immune surveillance (Brown et al., 2018). But, only a few studies have studied the influence of physical activity on cancer patient DCs, and further research is warranted in this regard.

#### 3.2.5 Platelets

Activation of platelets has an important role in enhancing the CTC survival rate. Once platelets are activated, they are CTC adhered, protecting cancerous cells from several different stress factors in the circulation. Furthermore, *in vitro* models show that CTC–platelet aggregates can inhibit anticancer cytotoxic effects of NK cells (Wang et al., 2022). It was shown that exercise altered the activity of platelets and CTC clearance (Wang et al., 2022). Physically active mice with breast cancer exhibited reduced platelets in circulation as compared to sedentary mice (Smeda et al., 2017). Moderate physical activity [60% maximal oxygen consumption (VO_2_max) for 40 min] in nasopharyngeal carcinoma patients reduced CTC–platelet aggregate formation and decreased metastasis risk (Wang et al., 1985). The CTC extravasation is also critically controlled by platelet activation. Activated platelets exhibited adhesion molecules to accumulate CTCs and efficiently bind to activated endothelial cells. Exercise can be linked to adhesion molecule reduction on endothelial cells and platelets like EPCAM-1 and P-selectin (Wang et al., 2005; Souza et al., 2017). P-selectin^−/−^ mice show significant reduction of lung metastases following tumor cell injection (Borsig, 2018). Nonetheless, intensity can be the rate-limiting factor for physical activity–mediated positive effects. Some studies report that high-intensity physical activity (<75% VO_2_max) led to CTC–platelet aggregation (Wang et al., 2022; Skouras et al., 2023), while moderate physical activity (50%–74% VO_2_max) prevented both adhesiveness and aggregation of platelets (Barale et al., 2023).

#### 3.2.6 T cells

Improving the functioning of T cells can be a useful therapeutic strategy in both hematologic and solid cancers. Some of the approaches include chimeric antigenic T cells, checkpoint inhibitors, adaptive T cell therapy, and the use of bispecific antibodies (Sharma and Allison, 2015; Velasquez et al., 2018). The use of common chemotherapy in cancer treatment worsens the already weakened T-cell function, causing severe depletion of T cells. Methods causing improvement in the functioning of T cells are of great importance, but there are several limitations to the existing approach, which include variable rate of response, associated toxicities, and economic burden to the healthcare system and patients, and hence alternate strategies are required (Desai and Gyawali, 2020). Studies have worked on the impact of physical activity on immunosenescence, a term associated with immune T-cell aging. An increase in the mortality risk has been linked to immunosenescence (Wikby et al., 1998; Caldeira et al., 2023). Immunosenescence features such as thymic output, defective senescent T cells, and vaccine responses can be improved through physical activity (Spielmann et al., 2011). A significant reduction in immunosenescence was observed in athletic individuals as compared to sedentary individuals (Duggal et al., 2018). Specifically, athletic individuals show improvements in both T-cell and thymic cell functions as they have higher thymic T-cell emigrants and CD41-positive naive T cells (Duggal et al., 2018). Additionally, the composition of immune T cells of both young healthy and highly athletic individuals was the same. *Ex vivo* T-cell (adenovirus-specific) production was improved through a cycling session above the normal lactate threshold level (Kunz et al., 2018). Likewise, a single physical activity session among young healthy adults elevated levels of T-cell cytotoxicity against tumor-specific antigens in preferentially expressed antigen in melanoma (PRAME) and melanoma antigen gene (MAGE) (LaVoy et al., 2015b). Therefore, both single-session and chronic aerobic physical activity training result in the improvement of T-cell functioning and open an alternate T-cell adoptive therapeutic strategy.

#### 3.2.7 B cells

Typically, following an acute session of aerobic exercise, B cells increase in numbers and then again decrease during recovery (Turner, 2016). Particularly, young healthy individuals show the greatest mobilization of immature B cells following acute aerobic physical activity. After physical activity, both naive (CD27neg/CD10neg) and memory (CD27pos/CD38neg) B-cell numbers were increased. B cells may be invigorated through acute aerobic physical activity, although it is a matter of ongoing investigation (Campbell and Turner, 2018). A study showing physical activity–mediated benefits on vaccine responses suggests an improvement in B-cell functioning as the production of B-cell immunoglobulin is crucial toward vaccine response (Simpson and Pawelec, 2021). Moreover, athletic individuals as compared to sedentary individuals show enhanced regulatory B-cell frequencies, which is critical in immune response regulation (Duggal et al., 2018). To summarize, a significant proportion of dysfunctioning immune cells that are either involved directly or in the cancer microenvironment get positively modulated in physically active individuals and therefore have the potential to positively influence malignancies as well.

### 3.3 Other effects of exercise on the immune system

Physical activity–mediated improvement in the functioning of the immune system is brought about partly by reducing the immune dysfunction–associated detrimental effects originating both as a result of obesity (immunosuppressive and inflammatory responses) and aging (inflammation and immunosenescence) (Stoner et al., 2016; Duggal et al., 2018; Salimans et al., 2022). However, physical activity also causes significant changes to adaptive and innate immune responses. In this section, we will discuss some of the other immunomodulatory effects exerted by exercise.

#### 3.3.1 Improved immunosurveillance

Since each bout of exercise results in a massive redeployment of lymphocytes, immune surveillance can be increased by acute aerobic physical exercise due to the redistribution and remobilization of the effector lymphocytes. Pedersen et al. (2016) demonstrated that tumor growth and incidence were decreased by 60% through wheel running in murine tumor models. The mechanism behind such tumor reduction was found to be dependent on catecholamine-driven redistribution and mobilization of NK cells (Pedersen et al., 2016). It is further suggested that tumors in exercised mice showed higher NK cell numbers that that in non-exercised mice, and this difference was not observable when propranolol was provided to the mice, which resulted in the prevention of catecholamine-driven mobilization of NK cells. Moreover, non-exercised mice showed a similar effect when they received daily epinephrine dosage, emphasizing the significance of catecholamines in inducing redistribution and mobilization of immune cells. Another study bolstered the idea that a single round of physical activity results in improving the immune surveillance of tumors through the observation that mobilization of T cells into circulation following acute aerobic physical activity showed a greater response to autologous antigen-presenting cell (APC) stimulation pulsed with antigens like PRAME, MAGE-A4, and Wilms tumor 1 (WT1) (LaVoy et al., 2015b). Chronic aerobic physical activity may exert the desired effect owing to the immune cells being redeployed and mobilized after every round of physical exercise, resulting in immune surveillance improvement without modifying the basal immune competency. Notwithstanding the fact that NK cell mobilization plays a critical exercise-mediated anti-cancerous effect (Pedersen et al., 2016), physical activity interventions show hardly any change in *in vitro* NK cell activity (Coletta et al., 2021). However, it has been reported that there is marked increase in NK cell function in individuals who show the greatest aerobic fitness changes after intervention (Coletta et al., 2021). It should be noted that most of the work describes the role of physical training on immune function endpoints that are “static” in nature (e.g., T-cell proliferation, NK cell cytotoxicity using resting blood cells). The adaptations in immune response may be observed better with endpoints that are “dynamic” like the number of T cells and NK cells that are mobilized following physical activity or these cells’ ability to infiltrate cancerous cells in mouse tumor models.

#### 3.3.2 Increased leukocyte mobilization

Acute aerobic physical activity (cycling, rowing, running) results in instant leukocyte mobilization to the blood circulation (Simpson and Pawelec, 2021). While mobilized cells are mainly constituted of granulocytes, the ratios of both monocyte to lymphocyte and granulocyte to lymphocyte are decreased, which is suggestive of exercise-mediated preferential recruitment of lymphocytes to the blood circulation (Simpson and Pawelec, 2021). Additionally, there is a relative change in lymphocyte subset mobilization such as of CD4^+^ T cells, B cells, CD8^+^ T cells, and (γδ) T cells (Rooney et al., 2018; Simpson and Pawelec, 2021). Again, there seems to be a preference among these cell types for those with characteristic enhanced potential for migration and differentiation (Simpson and Pawelec, 2021). Rapid egress kinetics is shown by lymphocytes, particularly with NK cells, notwithstanding the three- to five-fold increase during physical activity, reaching non-exercise levels following few minutes of end of exercise (Rooney et al., 2018). During the early exercise recovery phase, the active cytokine-secretory profile is shown by blood T cells and NK cells that exhibit elevated *in vitro* cytotoxic effect on certain cancer cell lines (LaVoy et al., 2015a; Bigley et al., 2015). Likewise, exercise-mobilized (γδ) T cells show greater proliferation upon stimulation with bisphosphonate antigens causing changes in the phenotype, promoting enhanced cytotoxic effect on several different tumor cell lines (Baker et al., 2019). The secretion of catecholamines, cytokines, and other hormones results in the priming, redistribution, and mobilization of stimulated effector lymphocytes due to acute aerobic physical activity. Studies suggest that this response can be exploited for therapeutic interventions like enhancing the immune response to vaccines or getting immune cell products from the blood with greater cellular therapy potential (Dhabhar, 2014; Simpson and Pawelec, 2021).

#### 3.3.3 The connection to myokines

Resting muscle has been found to be the source of nearly 300 secreted proteins that modulate different physiological processes (Stoner et al., 2016). Among these proteins are muscle-derived cytokines called myokines that provide beneficial effects on the immune function besides supporting myogenic growth and glucose metabolism. Interleukins such as IL-15, IL-8, IL-7, IL-6, and IL-4 are some of the prominent myokines that support the health of both the immune system and muscles (Stoner et al., 2016). The peripheral blood plasma IL-6 levels rise exponentially with enhanced duration and intensity of physical activity to approximately 100 times than the pre-exercise levels (Shephard, 2002). Although IL-6 shows both anti-inflammatory and pro-inflammatory properties, physical activity induces IL-6 to exert anti-inflammatory effects without inducing the usual pro-inflammatory context-dependent TNF-α increase (Kistner et al., 2022). Chronic aerobic physical activity causes a reduction in circulatory IL-6 levels (Kistner et al., 2022). The two cytokines IL-7 and IL-15 secreted highly by the skeletal muscle are important for T-cell homeostasis maintenance during exercise (Kim et al., 2008). They also work in coordination in replenishing memory and naive cell populations. IL-7 produces signals for the proliferation and survival of naive T cells and recent thymic emigrants while also supporting memory cell production and persistence after antigen exposure (Goldrath et al., 2002; Kim et al., 2008). Memory T cell particularly CD8^+^ expansion is supported by IL-15 (Kim et al., 2008). It has been found that both IL-7 and IL-15 promote cell survival through the upregulation of telomerase and anti-apoptotic activities. Proliferation can also be induced by cytokines through effector function acquisition and/or change in phenotype (Kim et al., 2008). IL-7 induces the renewal of a subset of CD8^+^ memory T cells, while this subset is expanded with the help of IL-15 (Cieri et al., 2013). The role of IL-15 is also crucial in NK cell expansion and differentiation (Pfefferle et al., 2020). It has been suggested that immunosenescence is developed as a result of reduced secretion from these cytokines. Altogether, it is generally accepted that muscle-derived cytokine stimulation results in supporting populations of effector immune cells by the maintenance of anti-inflammatory and pro-inflammatory mediator balance through precise homeostatic mechanisms.

## 4 Implications of exercise in clinical trials

Physical training is getting increasingly popular among investigators who are using it in clinical trials for improving response to anticancer therapeutic intervention, cardiorespiratory function, and the overall quality of life (Christensen et al., 2018; Iyengar and Jones, 2019; Koutoukidis et al., 2020; Quist et al., 2020). In aged cancer patients, physical activity can be used to inhibit immunosenescence (de Araujo et al., 2013). This concept of exploiting exercise oncology in augmenting cancer therapeutics has been advocated for some time now (Ashcraft et al., 2019; Holmen Olofsson et al., 2020). Although this field is relatively new, data from human studies and animal models lay the foundation for clinical trial designs on physical activity–driven immunotherapeutic interventions. For example, it has been shown in a study how to precisely look for the effect of physical activity in newly diagnosed lymphoma patients and observed that sedentary patients have lesser survival than active patients (Pophali et al., 2018). Similarly, it may be asked whether there is improvement in immune response with enhanced training programs such as aerobic capacity or physical activity. Several different concerns such as patient compliance, feasibility of physical activity incorporation into clinical trials, and ensuring safer training in frail patients have to be addressed for using exercise regularly in the cancer treatment setting. It is best to introduce physical activity in patients who have been newly diagnosed and have just started receiving treatment. Newer ways of patient compliance will be implemented with a better understanding of barriers to physical activity in cancer patients (Turner et al., 2018; Avancini et al., 2020). The major clinical studies that indicate the role of exercise as an adjuvant therapy in cancer treatment have been highlighted in Table 1.

**TABLE 1:** Studies showing the beneficial role of exercise in cancer therapy.

Serial No	Study	Year	Major findings
1	Quist et al. (2020)	2020	Lung cancer patients following an exercise regimen show improved muscle strength outcomes
2	Koutoukidis et al. (2020)	2020	Multiple myeloma patients responded safely to exercise with improved cardiovascular fitness and muscle strength
3	Rutkowska et al. (2019)	2019	Supervised exercise programs are beneficial in advanced lung cancer patients undergoing chemotherapy
4	Glass et al. (2015)	2015	In patients with solid tumors, aerobic training modulates select immune-inflammatory effector availability
5	Pophali et al. (2018)	2018	Lymphoma patients show improved overall survival with increased exercise following diagnosis
6	Hayes et al. (2018)	2018	Exercise intervention during and after treatment for breast cancer has survival benefit
7	Hornsby et al. (2014)	2014	Aerobic training when conducted under supervision is associated with improved patient reported outcomes and cardiopulmonary function during neoadjuvant chemotherapy
8	Andersen et al. (2006)	2006	Cancer patients undertaking a 6-week-long multidimensional physical activity intervention show reduced treatment-related symptoms while undergoing chemotherapy
9	Zylstra et al. (2022)	2022	Esophageal cancer patients receiving neoadjuvant chemotherapy show improved tumor regression when put under exercise intervention
10	Fairey et al. (1985)	2005	NK cell cytotoxicity is increased with exercise training

Clinicians are gaining access to published guidelines for incorporating exercise as part of the cancer patient treatment regime. Although making physical activity a part of clinical trials appears safe and feasible, there are patients with an inability to exercise. Another problem in the optimization of physical activity in cancer patient treatment is the lack of clarity about the physical exercise parameters that have the potential for disease outcome improvement. It is argued that single physical activity bouts are useful in cancer treatment, as physiological changes are brought about by exercise, even if transient in nature, that enhances the anticancer effects of exercise (Dethlefsen et al., 2017). However, considerable differences in the literature exist regarding cancer patient outcomes depending on physical exercise regimens. Cancer patients with shorter training periods do not seem to gain significant benefits in terms of their aerobic fitness, chemotherapy completion rates, and improved preoperative fitness (Mijwel et al., 2020). However, most cancer patients on an exercise regimen consistently show improvement in cardiorespiratory fitness (Hornsby et al., 2014). In the general population, there are reports suggesting changes in the immune system of athletes following training for short periods (Teixeira et al., 2014). Rheumatoid arthritis patients completing interval training show reduction in disease severity and circulating inflammatory monocytes and improved cardiorespiratory function (Bartlett et al., 2018). Exercise-dependent immunomodulation also enhances the efficacy of monoclonal antibody-mediated therapies against hematological cancers (Collier-Bain et al., 2023). In the future, data gathered on the impact of physical activity on cancer patient treatments will be critical as they will be useful in designing future studies to assess the significance of physical exercise on cancer immunotherapy response.

## 5 Conclusion and future perspectives

Existing studies suggest that moderate physical exercise can be beneficial in controlling cancer. The immune response is highly positive to physical activity, which in turn exerts inhibitory effects on tumor metastasis. During physical activity, a significant proportion of killer immune cells that show tumor cytotoxic effects get mobilized into the blood circulation. The mechanisms underlying the immunomodulatory effects of physical exercise are both diverse and extensive. However, there are ongoing efforts to explore precisely the mechanistic details behind exercise-driven benefits on immune response. It is noteworthy to mention that several critical aspects of the effect of physical exercise on immune response remain to be answered, and thorough investigations have to be performed before incorporating physical exercise routinely as part of an immunomodulatory intervention in cancer patients. Preliminary studies support the postulate that cancer patients on exercise regimens show better immunological fitness and response to therapeutic interventions. It is now important to perform systemic studies to test and validate these preliminary indications in clinical trials as there is significant evidence to initiate such studies going forward. This review tried to analyze the impact of physical exercise in modulating the immune response in cancer patients. With increased knowledge about the mechanisms of cancer preventative effects of exercise, it will be possible to design newer therapeutic strategies by incorporating physical exercise as an integral part of such interventions, which might contribute in providing overall cancer patient survival.
